# Evaluation of Atrial Electromechanical Delay to Predict Atrial Fibrillation in Hemodialysis Patients

**DOI:** 10.3390/medicina54040058

**Published:** 2018-08-25

**Authors:** Hakan Gunes, Abdullah Sokmen, Hakki Kaya, Ozkan Gungor, Murat Kerkutluoglu, Fatma Betul Guzel, Gulizar Sokmen

**Affiliations:** 1 Department of Cardiology, Sutcu Imam University, 46040 Kahramanmaras, Turkey; abdullahs27@yahoo.com (A.S.); drmuratkerk@gmail.com (M.K.); guliz58@yahoo.com (G.S.); 2 Department of Cardiology, Cumhuriyet University, 58140 Sivas, Turkey; drhakkikaya84@gmail.com; 3 Department of Nephrology, Sutcu Imam University, 46040 Kahramanmaras, Turkey; ozkan.gungor@yahoo.com (O.G.); fatmabetulduygu@hotmail.com (F.B.G.)

**Keywords:** atrial electromechanical delay, atrial fibrillation, hemodialysis

## Abstract

*Background and objective*: Prevalence of atrial fibrillation is higher in hemodialysis patients as compared to the general population. Atrial electromechanical delay is known as a significant predictor of atrial fibrillation. In this study, we aimed to reveal the relationship between atrial electromechanical delay and attacks of atrial fibrillation. *Materials and methods*: The study included 77 hemodialysis patients over 18 years of age giving written consent to participate in the study. The patients were divided into two groups based on the results of 24-h Holter Electrocardiogram (Holter ECG) as the ones having attacks of atrial fibrillation and the others without any attack of atrial fibrillation. Standard echocardiographic measurements were taken from all patients. Additionally, atrial conduction times were measured by tissue Doppler technique and atrial electromechanical delays were calculated. *Results*: Intra- and interatrial electromechanical delay were found as significantly lengthened in the group of patients with attacks of atrial fibrillation (*p* = 0.03 and *p* < 0.001 respectively). The optimal cut-off time for interatrial electromechanical delay to predict atrial fibrillation was >21 ms with a specificity of 79.3% and a sensitivity of 73.7% (area under the curve 0.820; 95% confidence interval (CI), 0.716–0.898). In the multivariate logistic regression model, interatrial electromechanical delay (odds ratio = 1.230; 95% CI, 1.104–1.370; *p* < 0.001) and hypertension (odds ratio = 4.525; 95% CI, 1.042–19.651; *p* = 0.044) were also associated with atrial fibrillation after adjustment for variables found to be statistically significant in univariate analysis and correlated with interatrial electromechanical delay. *Conclusions*: Interatrial electromechanical delay is independently related with the attacks of atrial fibrillation detected on Holter ECG records in hemodialysis patients.

## 1. Introduction

The prevalence of atrial fibrillation (AF) in patients with chronic renal failure undergoing hemodialysis is 2–3 times higher than the general population [1,2,3,4,5]. Hemodialysis patients are closely associated with the risk factors for the development of AF such as aging, hypertension, diabetes mellitus, coronary artery disease, and heart failure. Moreover, chronic renal failure and hemodialysis itself triggers development of AF by leading various metabolic and hemodynamic changes [6,7,8]. Other possible mechanisms that may explain the high prevalence of AF in hemodialysis patients include left atrial enlargement, left ventricular hypertrophy and activation of renin–angiotensin–aldosterone system [9,10,11,12,13,14].

Noninvasive echocardiographic markers are useful clinically to predict arrhythmias in various diseases. One of these echocardiograhic markers, electromechanical delay (EMD) that has been defined as the temporal delay between the detected onset of electrical activity and the realization of force in the myocardium is an indicator of atrial conduction heterogeneity and can be obtained easily by tissue Doppler imaging (TDI).It has been shown in previous studies that atrial EMD is a useful parameter to predict atrial arrhythmias in various diseases such as myotonic dystrophy type 1, generalized anxiety disorders, obesity-hypoventilation syndrome, and atrial septal aneurysms [15,16,17,18,19,20,21,22].

Recently, several studies evaluating the effects of hemodialysis on atrial electromechanical functions as compared to healthy control group have been reported [23,24,25]. However, relationship of atrial electromechanical functions with AF attacks in hemodialysis patients has not been documented yet. In this study, we investigated the relationship between atrial electromechanical delay and AF attacks in patients with chronic renal failure undergoing hemodialysis.

## 2. Material and Method

### 2.1. Study Population

This prospective cross-sectional study included 77 patients undergoing hemodialysis in Research Hospital of Medical Faculty, Kahramanmaras Sutcu Imam University, between February 2018 and May 2018. Inclusion criteria were determined as being older than 18 years of age and giving written consent to participate in the study. Exclusion criteria were congestive heart failure, presence of pacemaker, presence of bundle branch block on ECG, usage of antiarrhythmic medication influencing atrial conduction (beta blocker, digoxin), moderate to severe mitral and/or tricuspid regurgitation, presence of angina, documented and not revascularized severe coronary artery disease, active infections, hyperparathyroidism, hipo- or hyperthyroidism, and autoimmune diseases. The patients with known or documented previous AF (permanent, persistent, or paroxysmal) and the patients developing AF attacks during echocardiographic examination were also excluded from the study. Demographic and laboratory data of the patients were recorded. All patients underwent standard transthoracic echocardiography including tissue Doppler examination after hemodialysis. Patients also had 24 h Holter ECG monitoring starting on the morning following hemodialysis.

### 2.2. Holter ECG

The 3-Channel recordings with standard settings (Walk 400h Cardioline, Milan, Italy) were used for performing the 24 h Holter monitoring. An experienced cardiologist who had no information about the characteristics of patients evaluated the recordings gained from patients for the presence of <30-s-long or ≥30-s-long AF episodes. Supraventricular runs with >3 beats, lasting <30 s with absolutely irregular RR interval and no distinct p-waves were regarded as non-sustained AF. Self-terminating episodes having similar morphological characteristics and lasting ≥30 s were considered as paroxysmal AF

### 2.3. Standard Echocardiography

Experienced echocardiographers who had no information about the clinical details of each subject undertook transthoracic echocardiographic examinations using the Vivid 7^®^ cardiac ultrasonography system (GE VingMed Ultrasound AS; Horten, Norway) with 2.5- to 5-MHz probes. The left lateral and supine positions by 2D, M-mode, pulsed, and color flow Doppler echocardiography examination was conducted for each patient. Single lead electrocardiogram was continuously recorded. For all measurements, the average of at least three cardiac cycles was gained. European Society of Echocardiography guideline criteria was the basis for the examination of M-mode measurements and conventional Doppler echocardiographic examinations002E [26]. Doppler tracings and two-dimensional images were gained from parasternal long and short axes, apical and subcostal views. Measurement of left and right atrial dimension, end-systolic and end-diastolic dimensions of left ventricle (LV), diastolic LV posterior, and septal wall thicknesses was conducted. Measurement of left atrial volumes was done using the disc method and Simpson’s rule was used for estimating LV ejection fraction (EF). Mitral inflow velocities, namely peak E (early diastolic) and peak A (late diastolic), E/A ratio was preferred for evaluation of LV diastolic function and also deceleration time of the E-wave (DT) and isovolumic relaxation time (IVRT) was used.

### 2.4. Tissue Doppler Echocardiography (TDE)

**Figure 1:**
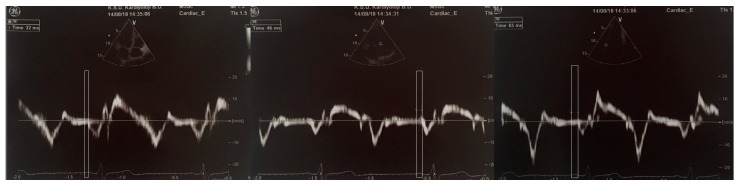
Measurement of time interval from the onset of P-wave on surface ECG to the beginning of late diastolic wave (Am wave) interval with tissue Doppler imaging (PA tricuspid, PA septal, and PA lateral, respectively).

The pulsed Doppler sample volume was placed at the level of LV lateral mitral annulus, septal mitral annulus, and RV tricuspid annulus from an apical four-chamber view. The time interval from the onset of the P-wave on surface ECG to the beginning of the late diastolic wave (Am), which is called PA, was gained from the lateral mitral annulus (PA lateral), septal mitral annulus (PA septal), and RV tricuspid annulus (PA tricuspid) (Figure 1). The difference between PA septum and PA tricuspid (PA septum − PA tricuspid) was identified as intra-atrial electromechanical delay while the difference between PA lateral and PA tricuspid (PA lateral − PA tricuspid) was identified as interatrial electromechanical delay [27,28,29].

### 2.5. Ethics Statement

The Kahramanmaras Sutcu Imam University Ethics Committee approved this study with protocol code 274. Each patient was enrolled after signing the informed consent. 

### 2.6. Statistical Analysis

The SPSS program version 14 (SPSS Inc., Chicago, IL, USA) was used for data management and analysis and a two-sided *p*-value ≤ 0.05 was found out to be statistically significant. Categorical variables were presented as the number of cases plus percentage and continuous variables as mean ± standard deviation (SD) or median and interquartile ranges (IQR), where applicable. Comparison of means was done through an independent sample *t* test, and, through a Mann–Whitney U test with median if there was no normal distribution. Using the chi square test was used if for appropriate for categorical data evaluation. Pearson correlation test was used for normally distributed variables and by Spearmen correlation test was used for non-normally distributed variables for correlation analysis was performance. Identification of the optimal cut-off point of inter- and intra-atrial EMD for the prediction of AF was done through receiver operator characteristic (ROC) curve analysis. ROC curve analysis was done using MedCalc (v12.7.8). The area under the curve (AUC) with 95% confidence interval was estimated in prediction of AF. The optimal cutoff value of interatrial EMD was identified as the value parallel with the highest sum of sensitivity and specificity-1. Univariate analysis was used for quantification of the association of variables with AF. The variables found out to be statistically significant in the univariate analysis and other potential confounders were used in multivariate logistic regression model with backward stepwise method for determining the independent prognostic factors of AF.

## 3. Results

Upon the analysis of Holter ECG recordings of 77 patients included in the study, 19 patients were detected to have AF attacks, while 58 patients were in sinus rhythm. Among patients with AF attacks, 7 of them had PAF and 12 of them had non-sustained AF. Clinical, laboratory and echocardiographic data of two groups formed according to the presence of AF were shown in Table 1. Hypertension ratio was significantly higher in the group developing AF attacks (*p* = 0.014), but there was no significant difference between groups regarding age, sex, and DM ratio. Among standard echocardiographic measurements, left atrial dimensions were significantly increased in the group with AF attacks (*p* = 0.01). Other standard echocardiographic and laboratory parameters were similar between two groups.

**Figure 2:**
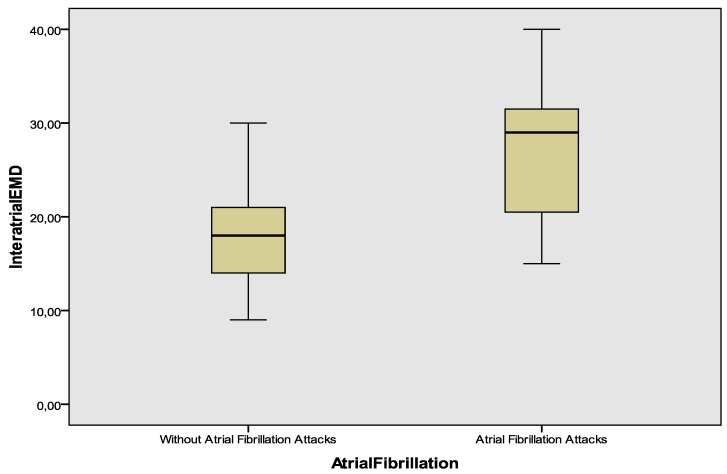
Distribution of interatrial electromechanical delay between the patients with and without AF attack. EMD = electromechanical delay.

Atrial electromechanical delay recorded from different annular segments were given in Table 2. Lateral and septal PA was significantly higher in the group developing AF attacks (64.9 ± 8.1 vs. 56.1 ± 7.4, *p* < 0.001; 47.4 ± 6.8 vs. 43.2 ± 6.6, *p* = 0.019 respectively). PA tricuspid was similar between groups. Inter- and intra-atrial EMD was significantly prolonged in the group with AF attacks as compared to the group in sinus rhythm (26.8 ± 7.3 vs. 17.7 ± 5.8, *p* < 0.001; 9 (4–14) vs. 3.5 (2–5.2), *p* = 0.04 respectively) (Figure 2).

It was found that interatrial EMD was positively correlated with intra-atrial EMD, septal PA, and lateral PA in all patients. Intra-atrial EMD was positively correlated with interatrial EMD, lateral PA, septal PA left atrial diameter, right atrial diameter, and TAPSE while it was negatively correlated with LVEF (Table 3).

Pearson correlation test was performed for normally distributed variables. Spearman correlation test was performed for non-normally distributed variables. *p* ≤ 0.05 was considered statistically significant

**Figure 3:**
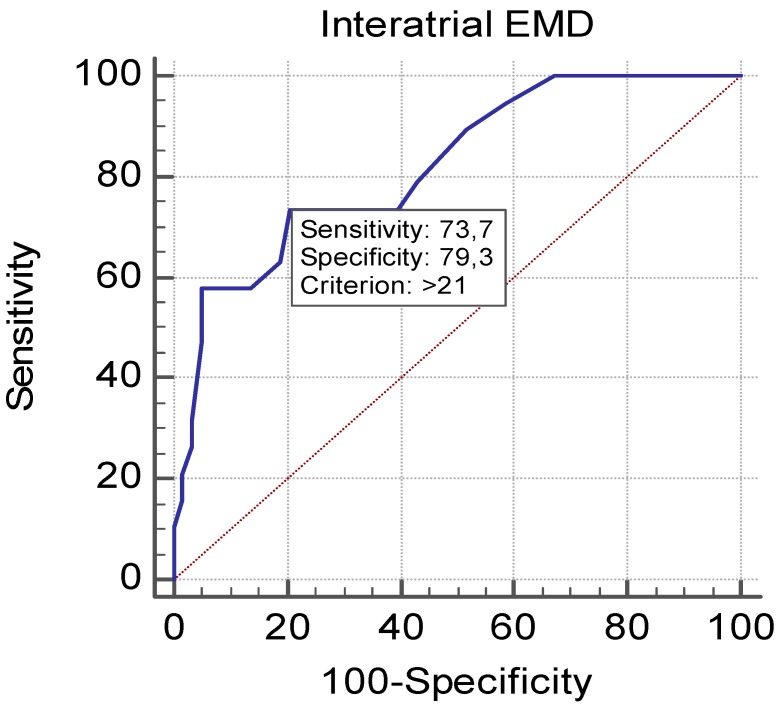
Receiver operator characteristic (ROC) Curve of Interatrial EMD to predict AF.

Optimal cut-off time of interatrial EMD to predict AF was found to be >21 ms, with specificity of 79.3% and sensitivity of 73.7% (AUC = 0.820; 95% CI, 0.716–0.898; *p* = 0.0001 (Figure 3).

This receiver operating characteristic curve shows that the optimal cutoff point of interatrial EMD in the prediction of AF was >21 ms, with specificity of 79.3% and sensitivity of 73.7% (area under the curve = 0.820; 95% CI, 0.716–0.898; *p* = 0.0001).

In the multiple logistic regression model using a back ward stepwise method, interatrial EMD (OR = 1.230, 95% CI: 1.104–1.370, *p* < 0.001) and hypertension (OR = 4.525, 95% CI: 1.042–19.651 *p* < 0.001) still remained significant predictors of AF after adjusting for the confounding variables, which were either found to be statistically significant in the univariate analysis and for the variables correlated with the inter- and intra-atrial EMD (Table 4).

## 4. Discussion

In this study, we showed that interatrial electromechanical delay was independently related to AF attacks detected by 24-h Holter ECG recordings in hemodialysis patients.

AF is more frequently seen in the patients on dialysis than normal population [30]. AF prevalence in dialysis patients varies between 7% and 27% [31,32,33]. VIVALDI (Vienna InVestigation of AtriaL Fibrillation and Thromboembolism in Patients on HemoDIalysis) study revealed that the prevalence of AF in these patients was 26.5%, of which 57.8% had paroxysmal AF, 3.0% had persistent AF, 32.5% had permanent AF and 6.6% had newly diagnosed AF [31,34]. In this study, we detected AF in 24% of the patients of whom 37% had paroxysmal AF, 63% had non-sustained AF. Although overall prevalence of AF in our study was similar to previous results, prevalence of AF subgroups was different from the ones reported in the literature. This difference might be explained by that duration of follow up was not long enough to make the diagnosis of persistent, permanent and long-standing AF. On the other hand, considering that non-sustained AF is a risk factor for the development of paroxysmal or permanent AF, or both, these ratios are expected to change during long-term follow up.

Of independent risk factors for the development of AF in general population, age, sex (male), hypertension, diabetes mellitus, heart failure, valvular heart diseases, and left atrial enlargement are also risk factors for the development of AF in dialysis patients [6,7,8]. In our study, the groups were similar to each other with regard to age, sex, and DM. Since many diseases that might affect atrial conduction properties such as coronary artery disease, valvular heart diseases, heart failure, and chronic inflammatory disorders were excluded, relatively small number and narrow spectrum of patients included in the study may be the reason of this indifference.

Hypertension contributes to development of AF through activation of renin angiotensin system that leads to changes in cardiac structure and physiology such as increased left atrial pressure and volume. In the Framingham study, hypertension was found as an independent risk factor for AF. Similar to previous reports in the literature, hypertension was found to be related with AF in hemodialysis patients on univariate and multivariate analysis in our study [35].

One of the possible mechanisms explaining the relationship between atrial dilatation and AF is decreased calcium flow through L-type calcium channels in enlarged atriums. The decrease in Ca flow shortens the action potential, and causes the loss of plateau phase in action potential and shortening of refractory period in atriums. It has been predicted that this electrical remodeling might be the etiology of AF. It may also cause electromechanical delay [36,37,38] Genovasi et al. have reported in their study including 488 chronic dialysis patients that left atrial enlargement is a risk factor for persistent and permanent AF, but not paroxysmal AF. In this study, paroxysmal AF developed in 8 of 202 patients with left atrial enlargement and in 9 of 286 patients without left atrial enlargement [31]. Similarly, we found that left atrial dilatation was not an independent risk factor for paroxysmal AF. We think that although electrical remodeling has started or developed, shown by increased electromechanical delay, structural remodeling playing role in the pathogenesis of AF such as myolysis, cellular hypertrophy, fibrosis, increased collagen type 1, increased activity of metalloproteinase may be at the beginning level or may not be progressed enough to cause atrial dilatation.

Electromechanical delay, EMD, is a parameter assessing the characteristics of atrial conduction which represents electrical and functional continuity of atrial myocytes, and is closely related to AF. EMD is formed by conduction velocity in atriums and atrial dimensions, and is an indicator of atrial hemodynamics. In congenital heart diseases, both chronic volume and pressure overload and postoperative scars in atriums decrease the velocity of atrial conduction and causes atrial electromechanical delay which has been shown to predict AF [39]. After myocardial infarction, fibrosis developing in atriums causes electromechanical delay by decreasing conduction velocity in atriums. Post-MI prolonged EMD is an independent risk factor for development of AF [40]. Histopathological changes in atrium are significant markers of EMD duration. Signs of structural remodeling such as atrial fibrosis, myocyte atrophy and scattered fibrotic fields in normal atrial tissue provide nonhomogenous impulse conduction in atriums. These signs are positively correlated with EMD. Because of these histopathological changes taking place in AF pathophysiology, both lone AF and recurrence of AF after cardioversion has been shown to be closely related with EMD [41,42,43]. Similarly, we showed that prolongation of EMD predicted AF in hemodialysis patients in whom atrial hemodynamic, functional, and electrical parameters are directly affected. 

Alterations of atrial hemodynamic parameters in hemodialysis patients, ongoing subclinical inflammation, RAAS activity, increased atrial pressure, and dilatation of atriums provide a basis for AF. These factors also lead to increase in EMD. Karavelioglu et al. showed that atrial EMD was increased in dialysis patients as compared to control group [44]. As different from previous studies, we detected AF attacks in Holter ECG recordings and documented that increased EMD predicted AF in these patients. Moreover, both inter- and intra-atrial EMD was prolonged in patients developing AF attacks, but only interatrial EMD was seen to predict AF on multivariate analysis. This may be explained by the fact that interatrial conduction involves both atriums and is affected by structural or electrical remodeling, or both, of any atrium. Determining the cut-off value of atrial EMD predicting AF in dialysis patients (21 ms in our study for interatrial EMD) may warn us to follow up the patients with measurements above the cut-off value closely and may be useful in early diagnosis and treatment of AF in such patients.

### Study Limitations

Main limitation of this study was that it was a cross-sectional study and the patients were not followed up for development of AF that was only detected by Holter ECG recordings. Holter ECG recordings were obtained for 24 h; prolonged or repeated Holter ECG monitorizations were not performed in non-sustained AF. Additionally, interobserver variability of PA measurements was not evaluated. Interatrial conduction time obtained by TDI was not correlated with invasive interatrial conduction time. Finally, relatively small number of subjects in the study and lack of long term follow up necessitates further studies to support our hypothesis.

## 5. Conclusions

To the best of our knowledge, this is the first study in the literature showing that interatrial conduction delay obtained by echocardiography is independently related to AF attacks detected on 24 h Holter ECG recordings in hemodialysis patients. Interatrial conduction delay, a noninvasive and easily obtained echocardiographic parameter, may be used to determine the dialysis patients who will benefit from 24 h Holter ECG monitorization to detect AF attacks.

## Figures and Tables

**Table 1:** Baseline characteristics of study patients.

Baseline Characteristics	All Patients (*n* = 77)	Without AF Attacks (*n* = 58)	AF Attacks (*n* = 19)	*p*
Age, mean ± SD, years	53 ± 15	54 ± 14	50 ± 17	0.362
Male/female, n	40/37	31/27	9/10	0.845
Hypertension, n (%)	32 (42%)	19 (32%)	13 (68%)	0.014
Diabetes mellitus, n (%)	25 (33%)	21 (36%)	4 (21%)	0.346
Heart rate, mean ± SD, beats/min	84 ± 15	84 ± 15	83 ± 16	0.726
Echocardiographic findings				
Left atrial diameter, mean ± SD, cm	3.7 ± 0.5	3.7 ± 0.5	4.0 ± 0.5	0.010
LV ejection fraction, mean ± SD, %	61 ± 5	61 ± 5	60 ± 5	0.671
LV end-systolic dimension, median (IQR), mm	33 (29–36)	33.5 (29.7–36)	32 (26–37)	0.423
LV end-diastolic dimension mean ± SD, mm	47.8 ± 5.1	47.9 ± 4.8	47.6 ± 6.3	0.868
RV diameter, mean ± SD, mm	29 ± 3.7	29 ± 3.8	29 ± 3.4	0.796
RV thickness, mean ± SD, cm	0.6 ± 0.1	0.6 ± 0.1	0.6 ± 0.2	0.884
Right atrial diameter, mean ± SD, mm	34 ± 5	34 ± 5	35 ± 4	0.276
RA end systolic area, mean ± SD, cm^2^	12.3 ± 3.9	12.4 ± 4.1	12 ± 3.1	0.768
RA end diastolic area median (IQR), cm^2^	18.1 (15.4–21.7)	18.5 (15.1–22.2)	18.1 (16.2–21.1)	0.976
TAPSE, mean ± SD, mm	28.4 ± 4.9	28.6 ± 4.7	27.6 ± 5.6	0.473
Posterior wall thickness median (IQR), mm	11 (11–12)	11 (11–12)	12 (11–13)	0.018
Septum thickness, median (IQR), mm	12 (12–14)	12.5 (12–14)	12 (11–15)	0.995
Mitral E velocity, mean ± SD, cm/sec	80.7 ± 24.5	81 ± 26.1	79.8 ± 19.4	0.842
Mitral A velocity, mean ± SD, cm/sec	87.2 ± 26.2	85 ± 26.9	93.4 ± 23.4	0.244
E/A, median (IQR)	0.86 (0.69–1.23)	0.88 (0.71–1.23)	0.80 (0.62–1.25)	0.233
Laboratory findings				
BUN, median (IQR), mg/dL	52 (42–63)	53.5 (60–63)	53 (50–62)	0.485
Creatinine, mean ± SD, mg/dL	7.4 ± 2.2	7.2 ± 2.3	7.9 ± 1.7	0.198
Triglycerides, median (IQR), mg/dL	175 (110–233)	182 (120–263)	144 (93–207)	0.167
Total cholesterol, mean ± SD, mg/dL	153 ± 37	153 ± 38	151 ± 37	0.834
HDL cholesterol, mean ± SD, mg/dL	36 ± 9	36 ± 10	37 ± 9	0.723
LDL cholesterol, mean ± SD, mg/dL	87 ± 31	88 ± 33	86 ± 26	0.853
Sodium, mean ± SD, mg/dL	139 ± 3	140 ± 3	138 ± 3	0.198
Calcium, mean ± SD, mg/dL	8.7 ± 0.7	8.7 ± 0.7	8.5 ± 0.5	0.196
Potassium, mean ± SD, mg/dL	4.9 ± 2.2	4.9 ± 0.7	5.0 ± 0.6	0.433

AF, Atrial fibrillation; BUN: Blood Urea Nitrogen, HDL: High-density lipoprotein, IQR: Interquartile ranges, LDL: Low-density lipoprotein, LV: Left ventricle, RV: Right ventricle, RA: Right atrium, TAPSE: Tricuspid annular plane systolic excursion; Data are presented as mean ± standard deviation (SD) number and percentage, or median and interquartile range (IQR). *p* ≤ 0.05 was considered statistically significant.

**Table 2:** Comparison of the Atrial Electromechanical Coupling Parameters Measured by Tissue Doppler Imaging.

	Without AF Attacks	AF Attacks	
	*n* = 58	*n* = 19	*p*
PA Lateral, mean ± SD, ms	56.1 ± 7.4	64.9 ± 8.1	<0.01
PA Septum, mean ± SD, ms	43.2 ± 6.6	47.4 ± 6.8	0.019
PA Tricuspid, mean ± SD, ms	38.3 ± 5.2	38.1 ± 4.1	0.827
Inter-atrial EMD, mean ± SD, ms	17.7 ± 5.8	26.8 ± 7.3	<0.010
Intra-atrial EMD, median (IQR), ms	3.5 (2–5.2)	9 (4–14)	0.04

PA = time interval from the onset of P-wave on surface ECG to the beginning of Am wave interval with tissue Doppler echocardiography, EMD = electromechanical delay, IQR: Interquartile ranges.

**Table 3:** Correlation Coefficients for Intra- and Inter-Atrial Electromechanical Delay.

	R	*p*
**Variables Correlating with Intra Atrial Electromechanical Delay**		
Inter-atrial EMD	0.596	<0.001
PA Septum	0.678	<0.001
PA Lateral	0.497	<0.001
Left atrial diameter	0.314	0.005
LV ejection fraction	−0.231	0.044
Right atrial diameter	321	0.005
TAPSE	252	0.027
**Variables Correlating with Inter-Atrial Electromechanical Delay**		
Intra-atrial EMD	0.586	<0.001
PA Septum	0.368	0.001
PA Lateral	0.810	<0.001

PA = time interval from the onset of P-wave on surface ECG to the beginning of Am wave interval with tissue Doppler echocardiography, EMD = electromechanical delay, TAPSE: Tricuspid annular plane systolic excursion.

**Table 4:** Univariate and multivariate analyses for predicting AF.

Variable	Univariate Analysis						Multivariate Analysis					
	B	S.E.	Wald	*p*	OR	95% Cl	B	S.E.	Wald	*p*	OR	95% Cl
**Statistically Significant Variables**												
Inter-atrial EMD	0.196	0.049	15.710	<0.001	1.216	1.104–1.339	0.215	0.56	14.905	<0.001	1.230	1.104–1.370
Hypertension	1.510	0.749	4.059	0.009	4.447	1.463–13.521	1.437	0.751	3.658	0.044	4.525	1.042–19.651
Left atrial diameter *	0.130	0.053	6.177	0.013	1.139	1.027–1.263						
PA lateral *	0.150	0.043	11.962	0.001	1.162	1.067–1.266						
PA septum *	0.095	0.042	5.099	0.024	1.100	1.013–1.194						
Intra-atrial EMD *	0.155	0.52	8.763	0.003	1.168	1.054–1.294						
**Variables Which Correlated with Intra-Atrial EMD**												
LV ejection fraction *	−0.021	0.049	0.186	0.666	0.979	0.889–1.078						
RA diameter *	0.059	0.054	1.198	0.274	1.061	0.954–1.180						
TAPSE *	−0.039	0.054	0.528	0.468	0.961	0.865–1.069						

All the variables from Table 1 and Table 2 were examined and only those significant at *p* < 0.05 level and correlated with inter- and intra-atrial electromechanical delay are shown in univariate analysis. Multivariate logistic regression analysis including all the variables in univariate analysis with enter method. *p* ≤ 0.05 was considered statistically significant. * Non-significant variables in multivariate logistic regression analysis were not indicated in the table. AF: Atrial Fibrillation; B: Beta coefficients; CI: Confidence interval; EMD = electromechanical delay. LV: Left ventricle, OR: Odds ratio, PA = time interval from the onset of P-wave on surface ECG to the beginning of Am wave interval with tissue Doppler echocardiography; S.E.: Standart Error; TAPSE: Tricuspid Annular Plane Systolic Excursion; RA: Right Atrium; Wald: Wald test.

## References

[1] Herzog C.A., Asinger R.W., Berger A.K., Charytan D.M., Díez J., Hart R.G., Eckardt K.U., Kasiske B.L., McCullough P.A., Passman R.S. (2011). Cardiovascular disease in chronic kidney disease. A clinical update from Kidney Disease: Improving Global Outcomes (KDIGO). Kidney Int..

[2] GBD 2013 Mortality and Causes of Death Collaborators (2015). Global, regional, and national age-sex specific all-cause and cause-specific mortality for 240 causes of death, 1990–2013: A systematic analysis for the Global Burden of Disease Study 2013. Lancet.

[3] Shiroshita-Takeshita A., Brundel B.J., Nattel S. (2005). Atrial fibrillation: Basic mechanisms, remodeling and triggers. J. Interv. Card Electrophysiol..

[4] Wetmore J.B., Mahnken J.D., Rigler S.K., Ellerbeck E.F., Mukhopadhyay P., Spertus J.A., Hou Q., Shireman T.I. (2012). The prevalence of and factors associated with chronic atrial fibrillation in Medicare/Medicaid-eligible dialysis patients. Kidney Int..

[5] Małyszko J., Bachorzewska-Gajewska H., Tomaszuk-Kazberuk A., Matuszkiewicz-Rowińska J., Durlik M., Dobrzycki S. (2014). Cardiovascular disease and kidney transplantation-evaluation of potential transplant recipient. Pol. Arch. Med. Wewn..

[6] Remppis A., Ritz E. (2008). Cardiac problems in the dialysis patient: Beyond coronary disease. Semin. Dial..

[7] Grande A. (2006). Atrial fibrillation and dialysis. A convergence of risk factors. Rev. Esp. Cardiol..

[8] Korantzopoulos P., Liu T., Li L., Goudevenos J.A., Li G. (2009). Implantable cardioverter defibrillator therapy in chronic kidney disease: A meta-analysis. EP Eur..

[9] Ananthapanyasut W., Napan S., Rudolph E.H., Harindhanavudhi T., Ayash H., Guglielmi K.E., Lerma E.V. (2010). Prevalence of atrial fibrillation and its predictors in nondialysis patients with chronic kidney disease. Clin. J. Am. Soc. Nephrol..

[10] Gupta J., Mitra N., Kanetsky P.A., Devaney J., Wing M.R., Reilly M., Shah V.O., Balakrishnan V.S., Guzman N.J., Girndt M. (2012). CRIC Study Investigators. Association between albuminuria, kidney function, and inflammatory biomarker profile in CKD in CRIC. Clin. J. Am. Soc. Nephrol..

[11] Bansal N., Keane M., Delafontaine P., Dries D., Foster E., Gadegbeku C.A., Go A.S., Hamm L.L., Kusek J.W., Ojo A.O. (2013). CRIC Study Investigators. A longitudinal study of left ventricular function and structure from CKD to ESRD: The CRIC study. Clin. J. Am. Soc. Nephrol..

[12] Levin A., Singer J., Thompson C.R., Ross H., Lewis M. (1996). Prevalent left ventricular hypertrophy in the predialysis population: Identifying opportunities for intervention. Am. J. Kidney Dis..

[13] Iravanian S., Dudley S.C. (2008). The renin-angiotensin-aldosterone system (RAAS) and cardiac arrhythmias. Heart Rhythm..

[14] Khatib R., Joseph P., Briel M., Yusuf S., Healey J. (2013). Blockade of the renin-angiotensin-aldosterone system (RAAS) for primary prevention of non-valvular atrial fibrillation: A systematic review and meta analysis of randomized controlled trials. Int. J. Cardiol..

[15] Dilaveris P.E., Gialafos E.J., Sideris S.K., Theopistou A.M., Andrikopoulos G.K., Kyriakidis M., Gialafos J.E., Toutouzas P.K. (1998). Simple electrocardiographic markers for the prediction of paroxysmal idiopathic atrial fibrillation. Am. Heart J..

[16] Omi W., Nagai H., Takamura M., Okura S., Okajima M., Furusho H., Maruyama M., Sakagami S., Takata S., Kaneko S. (2005). Doppler tissue analysis of atrial electromechanical coupling in paroxysmal atrial fibrillation. J. Am. Soc. Echocardiogr..

[17] De Vos C.B., Weijs B., Crijns H.J., Cheriex E.C., Palmans A., Habets J., Prins M.H., Pisters R., Nieuwlaat R., Tieleman R.G. (2009). Atrial tissue Doppler imaging for prediction of new-onset atrial fibrillation. Heart.

[18] Russo V., Papa A.A., Rago A., Ciardiello C., Nigro G. (2018). Effect of dual-chamber minimal ventricular pacing on paroxysmal atrial fibrillation incidence in myotonic dystrophy type 1 patients: A prospective, randomized, single-blind, crossover study. Heart Rhythm..

[19] Russo V., Rago A., Di Meo F., Papa A.A., Ciardiello C., Cristiano A., Calabrò R., Russo M.G., Nigro G. (2015). Atrial Septal Aneurysms and Supraventricular Arrhythmias: The Role of Atrial Electromechanical Delay. Echocardiography.

[20] Russo V., Di Meo F., Rago A., Mosella M., Molino A., Russo M.G., Nigro G. (2016). Impact of Continuous Positive Airway Pressure Therapy on Atrial Electromechanical Delay in Obesity-Hypoventilation Syndrome Patients. J. Cardiovasc. Electrophysiol..

[21] Russo V., Rago A., Papa A.A., Nigro G. (2017). Atrial fibrillation risk evaluation in patients with generalised anxiety disorders: The role of electrocardiographic parameters. Commentary to the article: “Atrial electromechanical delay analysed by tissue Doppler echocardiography”. Kardiol. Pol..

[22] Rago A., Russo V., Papa A.A., Ciardiello C., Pannone B., Mayer M.C., Cimmino G., Nigro G. (2017). The role of the atrial electromechanical delay in predicting atrial fibrillation in beta-thalassemia major patients. J. Interv. Card Electrophysiol..

[23] Demirtas L., Turkmen K., Buyuklu M., Kocyigit I., Orscelik O. (2016). Atrial electromechanical delay and left atrial mechanical functions in hemodialysis and peritoneal dialysis patients. Int. Urol. Nephrol..

[24] Tekce H., Ozturk S., Aktas G., Tekce B.K., Erdem A., Ozyasar M., Duman T.T., Yazici M. (2013). The effects of a single dialysis session on atrial electromechanical conduction times and functions. Kidney Blood Press Res..

[25] Turkmen K., Demirtas L., Topal E., Gaipov A., Kocyigit I., Orscelik O., Guney I., Kılıc S., Yilmaz M.I. (2015). Predictive Value of Atrial Electromechanical Delay on Long-Term Cardiovascular Outcomes in Hemodialysis Patients. Am. J. Nephrol..

[26] Lang R.M., Bierig M., Devereux R.B., Flachskampf F.A., Foster E., Pellikka P.A., Picard M.H., Roman M.J., Seward J., Shanewise J.S. (2005). Recommendations for chamber quantification: A report from the American Society of Echocardiography’s Guidelines and Standards Committee and the Chamber Quantification Writing Group, developed in conjunction with the European Association of Echocardiography, a branch of the European Society of Cardiology. J. Am. Soc. Echocardiogr..

[27] Acar G., Sayarlioglu M., Akcay A., Sokmen A., Sokmen G., Altun B., Nacar A.B., Gunduz M., Tuncer C. (2009). Assessment of atrial electromechanical coupling characteristics in patients with ankylosing spondylitis. Echocardiography.

[28] Bilgin M., Yıldız B.S., Tülüce K., Gül İ., Alkan M.B., Sayın A., İslamlı A., Efe T.H., Alihanoğlu Y.İ., Zoghi M. (2016). Evaluating functional capacity, and mortality effects in the presence of atrial electromechanical conduction delay in patients with systolic heart failure. Anatol. J. Cardiol..

[29] Sokmen A., Acar G., Sokmen G., Akcay A., Akkoyun M., Koroglu S., Nacar A.B., Ozkaya M. (2013). Evaluation of atrial electromechanical delay and diastolic functions in patients with hyperthyroidism. Echocardiography.

[30] Mlodawska E., Lopatowska P., Malyszko J., Banach M., Sobkowicz B., Covic A., Tomaszuk-Kazberuk A. (2018). Atrial fibrillation in dialysis patients: Is there a place for non-vitamin K antagonist oral anticoagulants?. Int. Urol. Nephrol..

[31] Genovesi S., Pogliani D., Faini A., Valsecchi M.G., Riva A., Stefani F., Acquistapace I., Stella A., Bonforte G., DeVecchi A. (2005). Prevalence of atrial fibrillation and associated factors in a population of long-term hemodialysis patients. Am. J. Kidney Dis..

[32] Fujii H., Kim J.I., Yoshiya K., Nishi S., Fukagawa M. (2011). Clinical characteristics and cardiovascular outcomes of hemodialysis patients with atrial fibrillation: A prospective follow-up study. Am. J. Nephrol..

[33] Vázquez-Ruiz de Castroviejoa E., Sánchez-Perales C., Lozano-Cabezas C., García-Cortés M.J., Guzmán-Herrera M., Borrego-Utiel F., López-López J., Pérez-Bañasco V. (2006). Incidence of atrial fibrillation in hemodialysis patients. A prospective long-term follow-up study. Rev. Esp. Cardiol..

[34] Königsbrügge O., Posch F., Antlanger M., Kovarik J., Klauser-Braun R., Kletzmayr J., Schmaldienst S., Auinger M., Zuntner G., Lorenz M. (2017). Prevalence of Atrial Fibrillation and Antithrombotic Therapy in Hemodialysis Patients: Cross-Sectional Results of the Vienna InVestigation of AtriaL Fibrillation and Thromboembolism in Patients on HemoDIalysis (VIVALDI). PLoS ONE.

[35] Schnabel R.B., Yin X., Gona P., Larson M.G., Beiser A.S., McManus D.D., Newton-Cheh C., Lubitz S.A., Magnani J.W., Ellinor P.T. (2015). 50 year trends in atrial fibrillation prevalence, incidence, risk factors, and mortality in the Framingham Heart Study: A cohort study. Lancet.

[36] Attuel P., Childers R., Cauchemez B., Poveda J., Mugica J., Coumel P. (1982). Failure in the rate adaptation of the atrial refractory period: Its relationship to vulnerability. Int. J. Cardiol..

[37] Le Grand B.L., Hatem S., Deroubaix E., Couétil J.P., Coraboeuf E. (1994). Depressed transient outward and calcium currents in dilated human atria. Cardiovasc. Res..

[38] Thiedemann K.U., Ferrans V.J. (1977). Left atrial ultrastructure in mitral valvular disease. Am J Pathol..

[39] Van der Hulst A.E., Roest A.A., Holman E.R., Vliegen H.W., Hazekamp M.G., Bax J.J., Blom N.A., Delgado V. (2012). Relation of prolonged tissue Doppler imaging-derived atrial conduction time to atrial arrhythmia in adult patients with congenital heart disease. Am. J. Cardiol..

[40] Antoni M.L., Bertini M., Atary J.Z., Delgado V., ten Brinke E.A., Boersma E., Holman E.R., van der Wall E.E., Schalij M.J., Bax J.J. (2010). Predictive value of total atrial conduction time estimated with tissue Doppler imaging for the development of new-onset atrial fibrillation after acute myocardial infarction. Am. J. Cardiol..

[41] Ari H., Ari S., Akkaya M., Aydin C., Emlek N., Sarigül O.Y., Çetinkaya S., Bozat T., Şentürk M., Karaağaç K. (2013). Predictive value of atrial electromechanical delay for atrial fibrillation recurrence. Cardiol. J..

[42] Spiecker M., Böhm S., Börgel J., Grote J., Görlitz S., Huesing A., Mügge A. (2006). Doppler echocardiographic prediction of recurrent atrial fibrillation following cardioversion. Int. J. Cardiol..

[43] Calık A.N., Ozcan K.S., Cağdaş M., Güngör B., Karaca G., Gürkan U., Yılmaz H., Bolca O. (2014). Electromechanical delay detected by tissue Doppler echocardiography is associated with the frequency of attacks in patients with lone atrial fibrillation. Cardiol. J..

[44] Karavelioğlu Y., Karapınar H., Özkurt S., Sarıkaya S., Küçükdurmaz Z., Arısoy A., Kurt R., Yılmaz A., Kaya M.G. (2014). Evaluation of atrial electromechanical coupling times in hemodialysis patients. Echocardiography.

